# High Efficiency *Ex Vivo* Cloning of Antigen-Specific Human Effector T Cells

**DOI:** 10.1371/journal.pone.0110741

**Published:** 2014-11-04

**Authors:** Michelle A. Neller, Michael H.-L. Lai, Catherine M. Lanagan, Linda E. O′Connor, Antonia L. Pritchard, Nathan R. Martinez, Christopher W. Schmidt

**Affiliations:** 1 Cancer Immunotherapy Laboratory, QIMR Berghofer Medical Research Institute, Brisbane, Queensland, Australia; 2 Oncogenomics Laboratory, QIMR Berghofer Medical Research Institute, Brisbane, Queensland, Australia; 3 School of Medicine, The University of Queensland Mayne Medical School, Brisbane, Queensland, Australia; New York University, United States of America

## Abstract

While cloned T cells are valuable tools for the exploration of immune responses against viruses and tumours, current cloning methods do not allow inferences to be made about the function and phenotype of a clone's *in vivo* precursor, nor can precise cloning efficiencies be calculated. Additionally, there is currently no general method for cloning antigen-specific effector T cells directly from peripheral blood mononuclear cells, without the need for prior expansion *in vitro*. Here we describe an efficient method for cloning effector T cells *ex vivo*. Functional T cells are detected using optimised interferon gamma capture following stimulation with viral or tumour cell-derived antigen. In combination with multiple phenotypic markers, single effector T cells are sorted using a flow cytometer directly into multi-well plates, and cloned using standard, non antigen-specific expansion methods. We provide examples of this novel technology to generate antigen-reactive clones from healthy donors using Epstein-Barr virus and cytomegalovirus as representative viral antigen sources, and from two melanoma patients using autologous melanoma cells. Cloning efficiency, clonality, and retention/loss of function are described. *Ex vivo* effector cell cloning provides a rapid and effective method of deriving antigen-specific T cells clones with traceable *in vivo* precursor function and phenotype.

## Introduction

In addition to their frequent use for *in vitro* studies of immune function, antigen-specific T cell clones are important tools for identifying viral and tumour antigens. They have also been expanded to large numbers for use in adoptive immunotherapy trials [1], [2]. The majority of T cell cloning methods involve stimulating unselected precursors for one or more rounds prior to limiting dilution cloning, to expand small populations of antigen-specific T cells [3], [4]. Whilst this procedure facilitates the isolation of rare T cells of interest, prior *in vitro* culture can have a number of undesirable effects. For example, the choice of cytokine combination, source of antigen and antigen dose can promote selective out-growth of particular T cell subpopulations [5]–[8] and affect the phenotype and function of the subsequently expanded cells. The effects of extended *in vitro* culture on T cell phenotype and function therefore preclude the correlation of many T cell clonal attributes with typical *in vivo* characteristics. Alternatively, T cells may be cloned directly *ex vivo*, by sorting individual T cells based on peptide-major histocompatibility complex multimer labelling [9], [10]; however, this requires knowledge of the epitope target, which prevents the use of such methods in antigen discovery or for generating clones against diverse virus or tumour antigens. A specialised technology for *ex vivo* cloning of HIV-Gag peptide-reactive CD8+ T cells, from arrays of sub-nanolitre wells that capture secreted cytokines, has also been described [11]. While this is at odds with the notion that effector cells have a limited potential for expansion in culture, as they are likely to be highly differentiated and possess short telomeres [12], [13], it suggests that *ex vivo* function could provide a basis for prior selection of T cells for efficient cloning.

We here describe a novel method for cloning effector T cells based on single-cell, fluorescence activated sorting of cytokine-secreting cells *ex vivo*. Direct cloning of antigen-specific effector T cells following a brief period of antigen stimulation, enables the acquisition of information on the characteristics of individual T cell clone precursors, prior to the influences of long-term culture, and repeated rounds of cell division. The method generates effectors with diverse specificities and HLA-restrictions by stimulating with complex antigen sources, such as whole tumour cells and whole protein, and enables the selection of T cells with known precursor *ex vivo* function and phenotype. By allowing the correlation of *ex vivo* T cell characteristics with more stable attributes (such as T cell receptor usage) identified for clones *in vitro*, this method adds a new dimension to the study of T cell responses to tumours and infections.

## Material and Methods

### Ethics Statement

The Queensland Institute of Medical Research - Human Research Ethics Committee approved this research under protocols P962 (approval H0609-044, cancer patients) and P598 (H0306-044, healthy donors), and all patients and donors gave written, informed consent prior to enrolment.

### Mononuclear cell isolation and cryopreservation

Peripheral blood from two Stage IV melanoma patients (both clinical trial participants, coded A02 and D14), and from healthy donors, was collected in tubes containing sodium heparin (BD Diagnostics, Franklin Lakes, NJ, USA). Peripheral blood mononuclear cells (PBMC) were isolated by density gradient centrifugation over Ficoll-Hypaque (GE Healthcare, Little Chalfont, UK) and cryopreserved in autologous plasma or Albumex 4 (Australian Red Cross Blood Service, Brisbane, Queensland) containing 10% dimethyl sulfoxide (Sigma-Aldrich Pty Ltd, St Louis, MO, USA). Cells were frozen in cryotubes (Thermo Fisher Scientific, Roskilde, Denmark), using Cryo 1°C Freezing Containers (Thermo Fisher), and cryopreservation was completed within 4 h of blood collection. Cells were thawed by incubating cryotubes in a 37°C waterbath, then cells were washed once in either RPMI 1640 (Life Technologies, Grand Island, NY, USA) containing 10 µg/ml DNase I (Sigma-Aldrich), for PBMC, or RPMI 1640 alone, for all other cell types.

### Cell culture media

Established cell lines were grown in complete medium: RPMI 1640 containing L-glutamine (Life Technologies) and supplemented with 10% heat-inactivated foetal bovine serum (Sigma-Aldrich) and 40 µg/ml gentamicin sulfate (Pfizer, Bentley, WA, Australia).

T cells were cloned and cultured in clone medium: RPMI 1640 containing L-glutamine, supplemented with 40 µg/ml gentamicin sulfate, 1 mM HEPES (4-(2-hydroxyethyl)-1-piperazineethanesulfonic acid), 100 IU/ml IL-2 (Roche Diagnostics GmbH, Mannheim, Germany) and 10% heat-inactivated pooled human serum (Australian Red Cross Blood Service). For initial sorting and restimulation, clone medium contained 1 µg/ml Phytohaemagglutinin-L (Sigma-Aldrich) and feeder cells as detailed below.

For functional assays, T cell clones and stimulator/target cells were suspended in assay medium – RPMI 1640 containing L-glutamine, 40 µg/ml gentamicin sulfate and 5% heat-inactivated pooled human serum or foetal bovine serum. All 37°C incubations were undertaken in 5% CO_2_.

### Cell lines

Melanoma cell lines (denoted A02-M and D14-M) were established from metastases from trial participants A02 and D14 [14], [15]. In brief, cells from mechanically disaggregated tumours were cultured in complete medium to establish cell lines, which were confirmed to be of melanoma origin by expression profiling [15], and authenticated via short tandem repeat profiling according to the manufacturer's instructions (AmpF‘STR Profiler Plus ID kit; Applied Biosystems, Foster City, CA, USA). B-lymphoblastoid cell lines (LCL) were established by exogenous transformation of peripheral B cells with Epstein Barr virus (EBV), derived from the supernatant of the B95.8 cell line, and were maintained in complete medium. The K562 cell line was obtained from the American Type Culture Collection (USA). All cell lines were tested negative for mycoplasma contamination using the Venor *GeM* Mycoplasma Detection Kit (Minerva Biolabs GmbH, Berlin, Germany) or the MycoAlert Detection Kit (Lonza Group Ltd, Basel, Switzerland), prior to use in experiments.

### Interferon (IFN)-γ capture assay and antibody labelling

The human IFN-γ secretion assay (phycoerythrin (PE) label; Miltenyi Biotec, Bergisch Gladbach, Germany) was used to detect antigen-specific T cells from patient PBMC samples. In each assay, 4-5×10^6^ PBMC were tested and a modification of standard protocols [16] was used. Donor/patient PBMC were thawed rapidly, washed, and resuspended in assay medium at 10^6^ cells per 200 µl well in U-bottom 96-well plates with 5×10^5^ irradiated (30 Gy) autologous LCL or melanoma cells. Alternatively, PBMC were stimulated with 1/100 (vol/vol) recombinant human cytomegalovirus (CMV) phosphoprotein 65 (pp65; Miltenyi Biotec). Cells were incubated at 37°C for 14 h (or as indicated in preliminary experiments), replicates were pooled, then washed with 0.5% bovine serum albumin/phosphate buffered saline (PBS) (FACS buffer), resuspended, transferred to capped 10 ml tubes and labelled with IFN-γ catch reagent, a CD45-specific monoclonal antibody (mAb) conjugated to an anti-IFN-γ mAb. The IFN-γ catch reagent was incubated with cells at a 1/10 dilution in a 50 µl total volume, for 15 min at 4°C. Cells were then resuspended at 1–2×10^4^ PBMC/ml in complete medium and incubated at 37°C for 1 h under slow rotation. For each stimulus, the optimal cell concentration for this step was determined empirically from the expected number of IFN-γ-secreting cells. Cells were subsequently washed twice with FACS buffer, and then labelled for 30 min at 4°C with pre-titred volumes of IFN-γ PE detection mAb (Miltenyi Biotec), CD8 allophycocyanin (APC; clone RPA-T8), CD4 Alexa Fluor 700 (RPA-T4), CD16 fluorescein (FITC; NKP15), CD19 FITC (HIB19), and CD14 FITC (MøP9) (BD Biosciences, Franklin Lakes, NJ, USA). Following a single wash with FACS buffer, cells were resuspended in 1 ml PBS containing 1 µg/ml propidium iodide (PI; Sigma-Aldrich).

### Sorting and cloning

**Figure 1:**
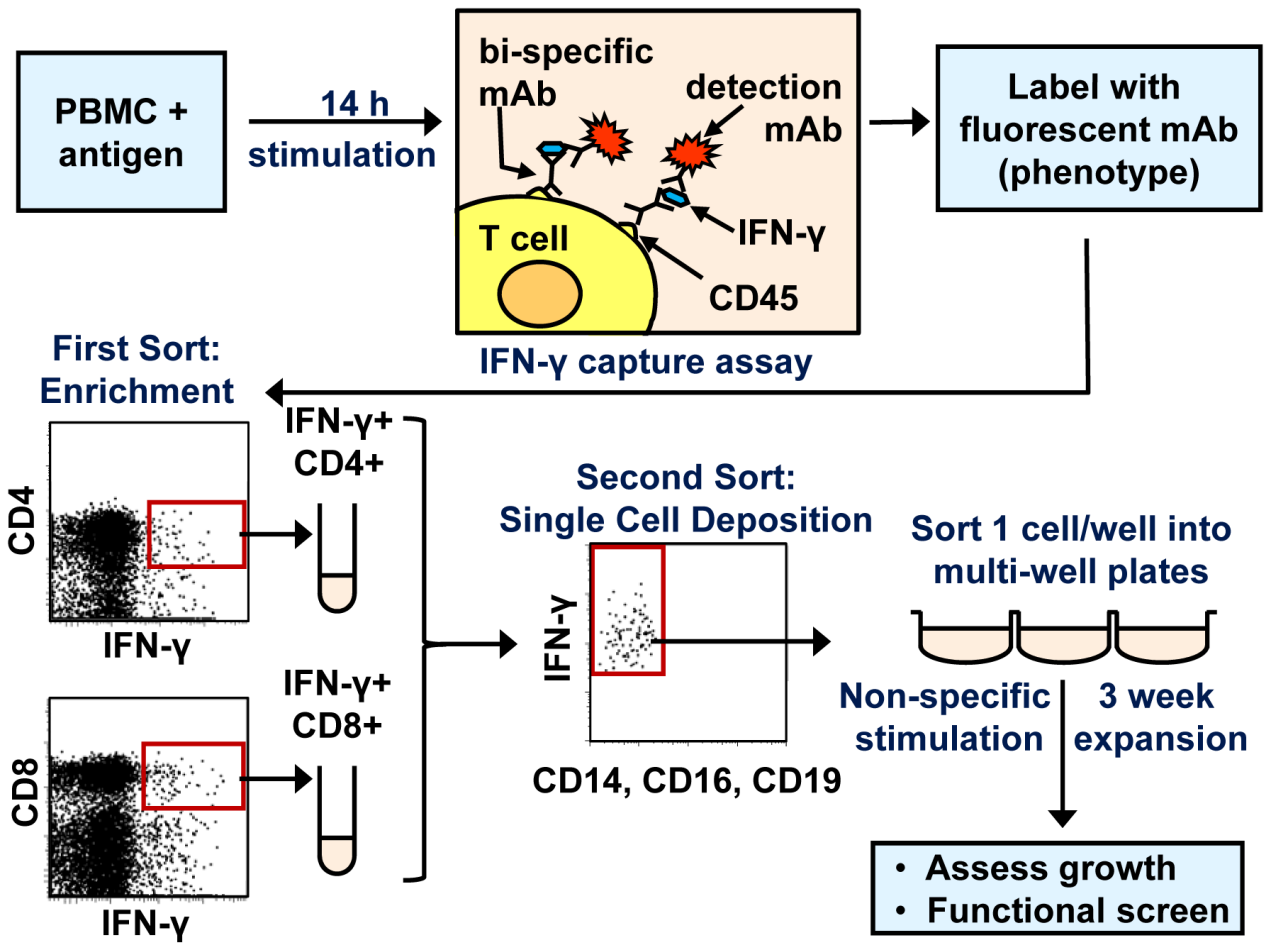
Overview of T cell clone generation. The procedure for cell stimulation, enrichment, single cell sorting, clone maintenance and characterisation is outlined. After PBMC were stimulated with antigen for 14 h, the IFN-γ capture assay was used to label functional cells. Cell phenotype and viability were revealed by the addition of fluorescent mAb against surface markers and propidium iodide. A MoFlo cell sorter enriched for viable, CD14– CD16– CD19– cells that were either CD4+ IFN-γ+ or CD8+ IFN-γ+. A FACS Vantage cell sorter then confirmed the phenotype of purified cell populations, and deposited functional cells into 96 well plates at one cell per well. Plates contained medium with feeder cells and phytohaemagglutinin-L, to non-specifically stimulate T cell clones. The growth and function of clones were assessed following expansion for three weeks, with weekly medium changes.

Cell suspensions were filtered through sterile 37 µm nylon mesh immediately prior to purification sorting of CD4+ IFN-γ+ and CD8+ IFN-γ+ populations using a MoFlo cell sorter running Summit software (Beckman Coulter, Fullerton, CA, USA). Sorting gates were determined by the bimodal expression of phenotypic markers (CD4, CD8, CD14, CD16, CD19) and IFN-γ, and in most cases were confirmed using negative controls. Subsequently a FACSVantage SE cell sorter running CellQuest and ClonCyt software (BD Biosciences) and equipped with a single cell deposition unit was used to sort single CD4+ or CD8+, IFN-γ+, CD14- CD16- CD19- cells into wells of U-bottom 96-well plates containing clone medium and feeder cells consisting of 2×10^4^ irradiated allogeneic LCL (a mixture of three different lines) and 1×10^5^ irradiated allogeneic PBMC per well. The overall cloning procedure is summarised in Fig. 1.

Every seven days, half the volume of medium in T cell clone plates (100 µl/well) was replaced with fresh clone medium. Proliferating clones could be visualised by microscopy after 10 days. Clones were re-plated in U-bottom 96-well plates at 5×10^4^ cells per well every four weeks and re-stimulated with clone medium containing phytohaemagglutinin-L and feeder cells.

### Flow cytometric T-cell receptor β variable (TRBV) chain analysis

To determine TRBV usage of T cell clones, aliquots of selected clones were labelled with a panel of 23 TRBV-specific mAb (currently available from Beckman Coulter). The T cell clones were incubated for 30 min at 4°C with CD8 APC, CD3 PE or FITC and one of the FITC- or PE-labelled TRBV-specific mAb. Cells were washed and analysed on a FACSCanto flow cytometer using FACSDiva software (BD Biosciences). Data were analysed and presented using FlowJo Software (Tree Star Inc., San Carlos, CA, USA).

### Cytotoxicity assay

The cytolytic activity of T cell clones was assessed in 4 h ^51^Cr-release assays. Autologous or allogeneic melanoma cells or LCL received fresh medium two days prior to use as targets. Target cells suspended in 50 µl assay medium were loaded with 50 µl Na_2_^51^CrO_4_ (PerkinElmer Inc., Waltham, MA, USA) for 1 h. ^51^Cr-labeled targets were then washed three times with RPMI 1640 and combined with effector cells at ratios ranging between 1∶10 and 1∶3 (10^3^ targets per well) in duplicate or triplicate in a total volume of 150 µl assay medium containing a 50× target excess of unlabelled K562 cells. Maximum ^51^Cr release was defined by incubation of targets with assay medium containing 0.1% sodium dodecyl sulfate (Sigma-Aldrich), and spontaneous ^51^Cr release was determined by incubation of targets with assay medium alone. Plates were centrifuged at 50 g for 2 min, and then incubated for 4 h at 37°C. After incubation, 25 µl of supernatant was removed from each well, transferred to LumaPlates (Packard Bioscience, Lenexa, KS, USA) and allowed to dry overnight, prior to measurement as counts per minute (cpm) ^51^Cr release using a TopCount Microplate Scintillation Counter (Packard). Percent specific lysis was calculated using the standard equation:
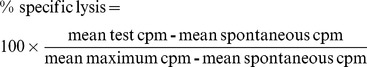



Clones were considered to be specific if they lysed autologous targets at>15% of maximum lysis, and lysed allogeneic targets <5%.

### IFN-γ ELISA

Antigenic stimulation was provided by autologous or allogeneic LCL or melanoma cells, or irradiated autologous LCL that had been pre-incubated with recombinant CMV pp65 (1 µl protein/100 µl assay medium/10^6^ cells) for 1–2 h. T cell clones were combined with stimulator cells at ratios of 50∶1 or 10∶1 (as detailed in figure legends). After 20 h, 100 µl supernatant was removed from each well to measure IFN-γ secretion by ELISA, using standard methods (Mabtech AB, Stockholm, Sweden) in PBS. Standard curves and cytokine concentrations were calculated using SOFTmax PRO software (Molecular Devices, Sunnyvale, CA). Clones were considered to be specific if they produced>100 pg/ml IFN-γ above controls (allogeneic LCL, allogeneic melanoma cells, or autologous LCL without CMV pp65).

### Proliferation assay

CD4+ clones derived from EBV antigen stimulation were combined in assay medium with gamma-irradiated (150 Gy) autologous LCL at a stimulator: responder ratio of 1∶20 in duplicate wells of a U-bottom 96 well plate. Proliferation was assessed by addition of 1µCi/well ^3^H-thymidine (Amersham Pharmacia Biotech Pty Ltd, Australia) for the last 6 h of the 3 day culture period. Cells were harvested onto glass fibre filter mats and ^3^H incorporation measured as cpm on a MicroBeta scintillation counter (Wallac, Finland).

### One-step TRBV gene sequence analysis

*TRBV* sequence analysis of T cell clones was undertaken using an adaptation of a previously described method [17]. RNA was extracted from 10^5^ cells, using PureZOL RNA isolation reagent (Bio-Rad Laboratories, Hercules, CA, USA). RNA pellets were suspended in 20 µl of diethyl pyrocarbonate-treated deionised water (MP Biomedicals Inc., Solon, OH, USA). RNA was reverse transcribed and amplified in a one-step reverse transcription-PCR (RT-PCR) system (Life Technologies). Primers used in one-step reactions and subsequent sequencing reactions are as follows: Degenerate forward primers VP1 (5′-GCIITKTIYTGGTAYMGACA-3′) and VP2 (5′-CTITKTWTTGGTAYCIKCAG-3′), specific forward primers VP3 (5′-ATCCTTTATTGGTATCGACGT-3′) and VP4 (5′-ATGTTTACTGGTATCATAAG-3′), specific reverse primer CP1 (5′-GCACCTTCCTTCCCATTCAC-3′). Each initial 25 µl reaction contained 12.5 µl of 2× reaction mix, 0.5 µl RT/Platinum Taq mix, 1 µl RNA, 200 nM CP1 and 2 µM VP1. If no product was detected in the PCR product by agarose gel electrophoresis, the one-step reaction was repeated using CP1 (200 nM), VP2 (2 µM), VP3 and VP4 (200 nM). RT-PCR cycling was performed at 50°C for 30 min and 94°C for 2 min. PCR cycling was then performed at 94°C for 20 s, 50°C for 40 s then 72°C for 40s for 40 cycles, with a final 10 min extension at 72°C. International ImMunoGeneTics information system (IMGT) nomenclature is used throughout this report [18].

### Sequencing of PCR products

PCR products were sequenced with BigDye Terminator v3.1 Cycle Sequencing Kit (Applied Biosystems, Foster City, CA, USA), according to the manufacturer's protocol, using a VP1 or VP2 primer. Sequences were then analysed on an ABI Prism 310 Genetic Analyzer (Applied Biosystems Inc., Foster City, CA, USA). Product sequences were assessed using Chromas software (Technelysium Pty Ltd, Tewantin, Qld, Australia). *TRBV(D)J* usage was determined using the IMGT V-QUEST online analysis tool [19].

### Statistics

Standard statistical tests (99% confidence intervals, standard errors of the mean, Kolmogorov-Smirnov test for differences in data distribution) were performed and graphs created in Prism v6.02 (GraphPad Software, San Diego, CA, USA).

## Results

### Optimisation of T cell stimulation

**Figure 2:**
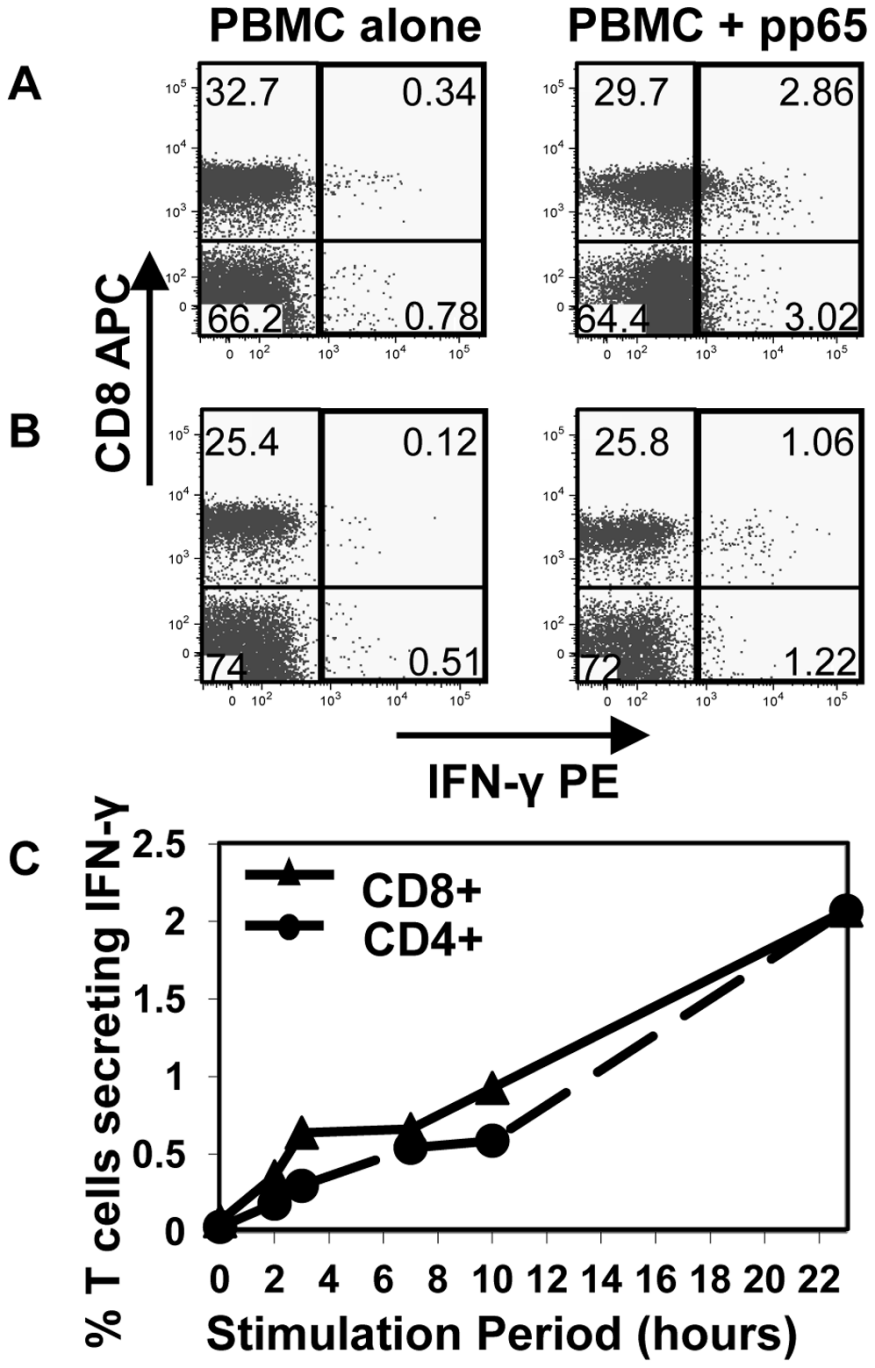
Optimisation of IFN-γ capture assay. (A, B) PBMC from healthy donors were stimulated for 14 h with recombinant CMV pp65, or (C) for increasing periods of time with the autologous lymphoblastoid cell line (LCL), then assessed for reactivity by IFN-γ capture assay. Cells were maintained at (A) too high a concentration during IFN-γ secretion, or (B) an optimal concentration. Cells are gated on viable, CD14– CD16– CD19– subsets. (C) The percentage of CD8+ and CD4+ T cells secreting IFN-γ, gated on viable CD3+ cells. Results are representative of (A, B) two, or (C) three independent experiments.

The human IFN-γ secretion assay (Miltenyi Biotec) uses cytokine capture to identify viable, functional T cells responding to antigenic stimulation. This kit has been used to purify IFN-γ-secreting cells prior to cloning by limiting dilution [20], but we wanted to determine if it could be combined with single-cell sorting to clone effector T cells *ex vivo*. Non-specific staining of bystander T cells in close proximity to cytokine-secreting effectors (“cross-feeding”) is a well recognised problem with the cytokine capture technique. In preliminary tests of the IFN-γ detection kit, a shift in the IFN-γ negative population was occasionally observed, despite following the manufacturer's recommendations (Fig. 2A). By decreasing the concentration of cells during the one hour IFN-γ secretion period to 1–2×10^4^ PBMC/ml, the negative population consistently remained in the left quadrants defined by stained, unstimulated PBMC (Fig. 2B).

The period of stimulation prior to IFN-γ capture was also varied, to identify a timepoint at which IFN-γ-secreting T cells could be detected prior to significant antigen-specific proliferation. Although responses were detected as early as 45 min in some analyses, extensive longitudinal experiments consistently showed that 10-23 h maximised responses (Fig. 2C). In subsequent experiments, PBMC were stimulated for 14 h, a convenient period minimising the possibility of *in vitro* division prior to sorting, and maximising the number of cells detected.

### Identification and sorting of antigen-specific effector T cells

**Figure 3:**
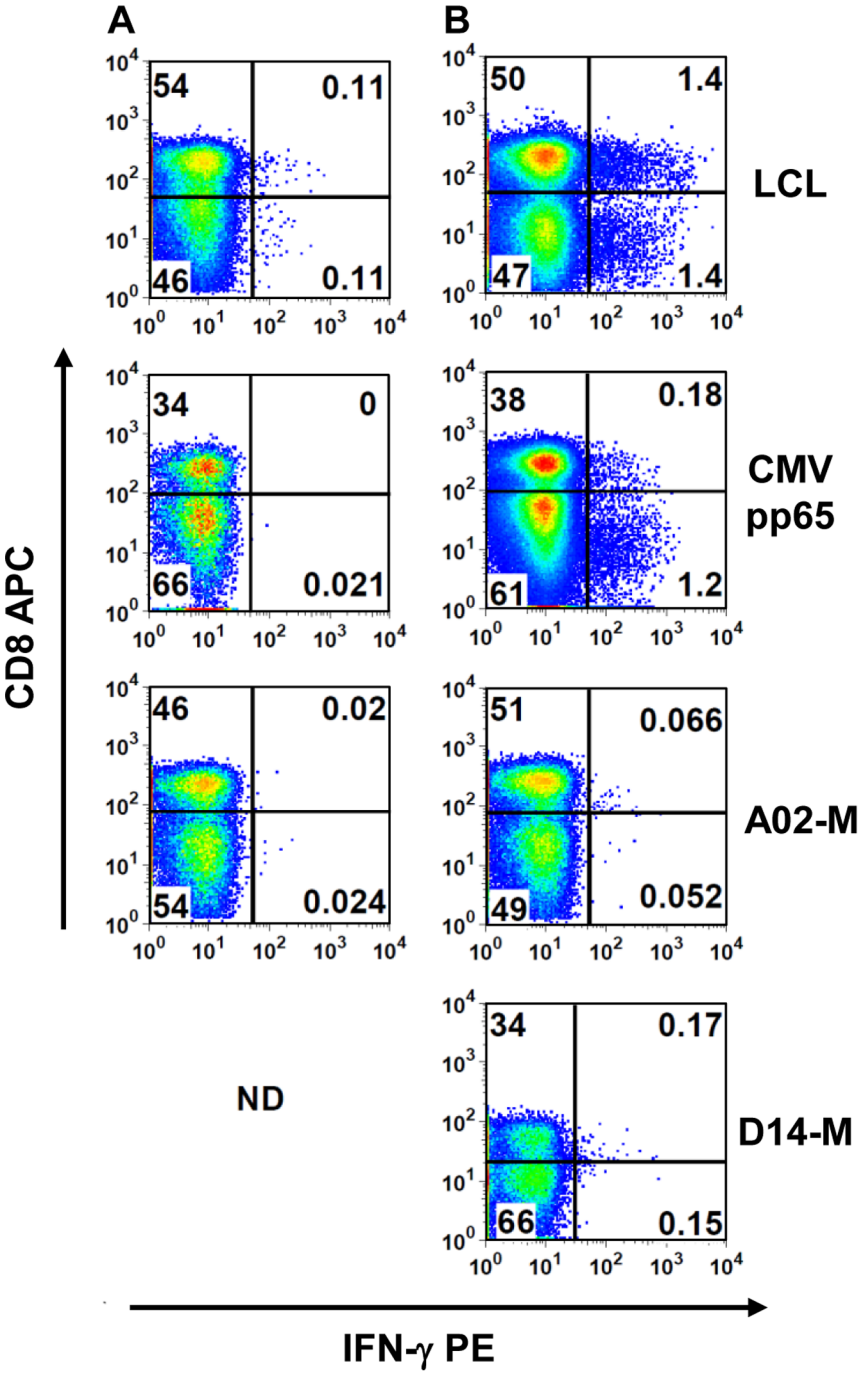
Detection of antigen-specific effector T cells *ex vivo*. IFN-γ production measured by the capture assay in (A) unstimulated PBMC samples and (B) PBMC from healthy donors stimulated with the autologous lymphoblastoid cell line (LCL) or CMV pp65 (top two panels), or PBMC from melanoma patients A02 or D14 stimulated with autologous melanoma cells (bottom two panels), as indicated. Cells were gated on the single cell, low scatter, viable, CD14–CD16–CD19– subpopulation. Percentages are shown in each quadrant; ND  =  not done.

Current T cell cloning methods frequently require prior, antigen-driven cell expansion, to overcome low precursor frequencies. However, in chronic infections, antigen-specific effector cell frequencies are elevated. Initial experiments indicated that IFN-γ production by T cells stimulated with autologous cell lines presenting cancer or viral antigens could be detected using flow cytometry, suggesting that *ex vivo* sorting based on function could enrich for antigen-specific cloning. In four separate experiments, different antigen sources were used to stimulate antigen-specific T cells: autologous melanoma cells (for two different patients), autologous LCL, or recombinant CMV pp65. PBMC from each donor were thawed, stimulated for 14 h with a single antigen source, and then IFN-γ-secreting T cells were identified using the cell surface capture IFN-γ detection kit. As expected, the magnitude of the responses to each antigen source varied, with IFN-γ-secreting T cells ranging from 0.1% of all T cells from melanoma patient A02 to 2.8% in the EBV model (Fig. 3). Although *ex vivo* T cell responses were very low for melanoma cell-stimulated PBMC, they were at least 2 fold higher than backgrounds detected in unstimulated PBMC from each donor. In summary, in each case precursor frequencies were sufficient to detect and sort antigen-reactive effector T cells based on phenotype and IFN-γ secretion.

### Cloning virus- and tumour-specific CD8+ and CD4+ T cells

Using the method outlined in Fig. 1, T cells specific for viral and tumour antigens were identified and sorted in two steps: following a preliminary enrichment for CD4+ IFN-γ+ and CD8+ IFN-γ+ T cells using a MoFlo cell sorter, single IFN-γ+ cells were then deposited into individual wells of a 96 well plate using a FACS Vantage SE cell sorter. This two step, tandem procedure eliminated the need to re-configure the primary sorter for single cell deposition. In four separate experiments, 26-79% of single-sorted T cells proliferated as clones after three weeks in culture (Table 1). CD4+ and CD8+ T cells from the healthy donors (stimulated with CMV pp65 or autologous LCL) and melanoma patient A02 (stimulated with the autologous melanoma cell line) cloned at comparable efficiencies (26-47% of all wells grew). However, CD8+ T cells from melanoma patient D14 had a substantially higher cloning efficiency, as all wells proliferated initially, and 79% of the sorted T cells generated long term clones. No relationship was found between cloning efficiency and precursor frequency, as PBMC from patient D14 had one of the lowest *ex vivo* responses to antigenic stimulation. The subsequent growth of a subset of wells seeded with CD8+ T cells producing IFN-γ following CMV pp65 stimulation was assessed, using the “index sorting” function available in ClonCyte software. A significantly different distribution of IFN-γ production was observed between wells that grew *vs*. those that died (P<0.0001; Kolmogorov-Smirnov test), consistent with a lower cloning efficiency for cells producing low amounts of IFN-γ *ex vivo* (Fig. S1). This suggests that, in this instance, the fitness of CD8 T cells for subsequent expansion was related to their functional response to cognate antigen.

**Table 1:** Cloning efficiency and functional retention of T cell clones.

Stimulus	T cell Subset	Wells Seeded	Established Clones^a^	IFN-γ+ clones^b^	Functional clones^d^
LCL	CD4+	234	62 (26%)	5/5^c^	15/65 (23%)
	CD8+	246	116 (47%)	9/107 (8%)	15/126 (12%)
CMV pp65	CD4+	250	87 (35%)	41/87 (47%)	–
	CD8+	230	66 (29%)	19/66 (29%)	–
A02-M	CD4+	114	32 (28%)	19/31 (61%)	–
	CD8+	162	57 (35%)	23/63 (37%)	23/63 (37%)
D14-M	CD4+	ND	ND	ND	ND
	CD8+	288	227 (79%)	221/288 (77%)	267/288 (93%)

a Number of growing clones, percentage of wells seeded in brackets.

b Number of clones producing>100 pg/ml more IFN-γ in response to autologous/antigen loaded autologous targets than to control targets, as a fraction of total clones tested; percentage in brackets. In some cases clones that did not proliferate long term were tested, so the denominator is different to the number of established clones.

c CD4+ clones from LCL stimulation were pre-selected from a subset which were positive for antigen-stimulated proliferation, and thus may not be representative of the 62 proliferating clones.

d Clones were tested for cytolytic activity (CD8+ clones from LCL and melanoma stimulation only), IFN-γ production (CD4+ and CD8+ clones from each stimulation), or proliferation (CD4+ LCL clones only). Data represent number of clones with any significant response to autologous antigen, as a fraction of total number of clones tested.

ND, not done; – indicates no test additional to IFN-γ production was performed.

Clones were re-stimulated with mitogen every four weeks, and using this procedure, it was possible to expand clones to obtain up to 10^8^ cells per clone, after repeated stimulation.

### Functional assessment of T cell clones

The use of T cell clones is generally dependent on their expression of some antigen-specific function. We therefore analysed clones for functional characteristics in response to antigenic stimulation, given that effector T cells can alter their expression of cytokines following expansion [21].

**Figure 4:**
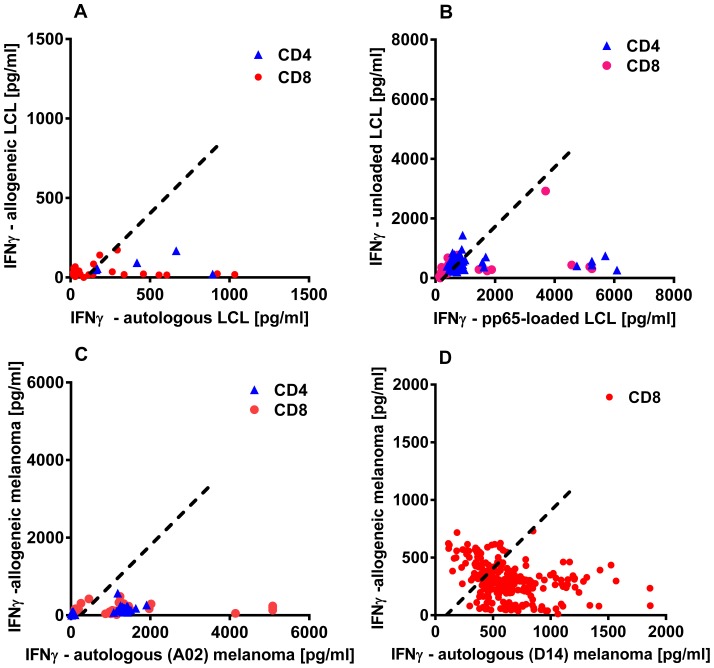
Retention of IFN-γ production by T cell clones. ELISA was used to measure IFN-γ in supernatants of T cell clones (▴CD4+; • CD8+) stimulated with (A) autologous or allogeneic lymphoblastoid cell lines (LCL), (B) autologous LCL either unloaded or pre-incubated with recombinant pp65 for 1-2 h, or (C, D) autologous or allogeneic melanoma cells (as indicated in graph). The stimulator: T cell responder ratios were (A–C) 50,000∶1,000 or (D, patient D14) 100,000∶10,000. Dotted line indicates>100 pg/ml in excess of control, the cut-off for positivity used in Table 1, which summarises the data from this figure.

CD4+ and CD8+ clones were stimulated with autologous or allogeneic melanoma cells, LCL or LCL loaded (or untreated) with CMV pp65 protein, as appropriate, and then culture supernatants were analysed for IFN-ã by ELISA (Fig. 4 A–D). The proportion of clones that retained IFN-γ production varied from 8% (CD8+ clones derived from LCL stimulation) to 77% (CD8+ clones from melanoma patient D14; Table 1). As with cloning efficiency, the ability of clones to secrete IFN-γ was not related to higher precursor frequency – indeed, the opposite may be true, as the high proportion of cells producing IFN-γ *ex vivo* in response to CMV and EBV translated into lower proportions of functional CD8+ T cells (Table 1).

**Figure 5:**
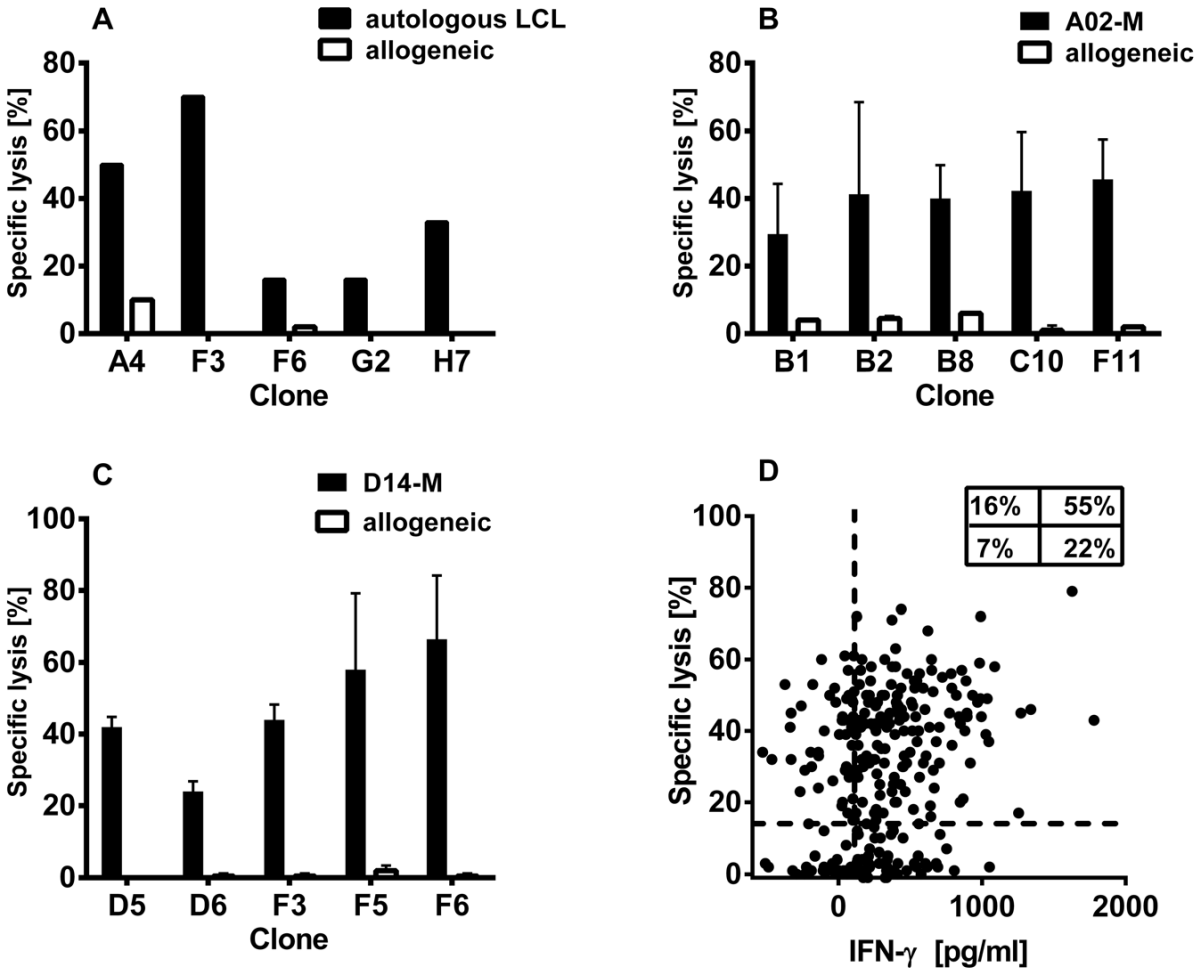
Cytotoxicity of CD8+ T cell clones. Cytotoxicity of selected CD8+ T cell clones from (A) healthy donor, (B) melanoma patient A02, and (C, D) melanoma patient D14, was assessed against the autologous and allogeneic lymphoblastoid cell line (LCL) or melanoma cells by chromium release assay at an effector: target ratio of 10∶1. Data indicate the mean specific lysis from (A) one; (B) three; and (C) two independent experiments, and error bars represent the standard error of the mean. (D) T cell clones from patient D14 were simultaneously assessed for IFN-γ production in response to autologous and allogeneic melanoma cells (as described in Fig 4). The specific lysis of autologous melanoma cells (vertical axis) is plotted against IFN-γ production (data corrected by subtracting allogeneic release). Dotted lines indicate cut-offs for positivity (see methods); inset indicates percentages positive in each quadrant.

To determine whether T cells sorted on the basis of IFN-γ production were capable of killing, selected LCL- and melanoma-stimulated CD8+ clones were tested for cytolytic activity against autologous and allogeneic cell lines. Strong activity was demonstrated for all tested CD8+ clones (Fig 5 A, B, C), with minimal reactivity against the allogeneic control. To obtain representative data across a broader range of clones, we screened all patient D14 CD8^+^ T cell clones against autologous and allogeneic melanoma lines using ^51^Cr-release assays (Fig 5D). Cytolytic activity was demonstrated for 75% of clones, of which 16% were scored negative for IFN-γ production in the parallel assay (Fig 5D). Likewise, a proportion of clones secreted IFN-γ but lacked cytolytic activity against the autologous melanoma line. Overall, only 7% of sorted, proliferating D14 CD8+ T cell clones failed to exhibit significant functional activity in either of these assays.

Selected CD4+ clones derived by LCL stimulation of healthy donor PBMC were also tested for proliferation in response to autologous antigen using a tritiated thymidine assay; a total of 14/63 clones proliferated significantly above background defined by non-stimulated controls (Figure S2).

Overall, between 12% and 93% of clones expressed some functional response to antigen stimulation. This is likely to be an underestimate of the true total for some stimuli, as (except for patient D14) only selected clones were tested for cytolytic activity or proliferation (Table 1).

### Assessment of clonality by TRBV analysis

Clonality is required for many applications, for example, antigen discovery and studies of T cell receptor usage. The most common method of T cell cloning, limiting dilution, relies on the statistical probability that no more than one proliferating cell will be placed in each well during plate setup. This makes the method inefficient, as many wells will not contain T cells, and re-cloning is often required due to outgrowth of mixed populations. Cloning using single cell sorting avoids this problem, by providing a definitive method of obtaining clonal T cells, whilst employing comparatively few plates. The only confounding factor would be if cross-contamination occurred during sorting, or during extended culture, which could compromise the outcome.

**Figure 6:**
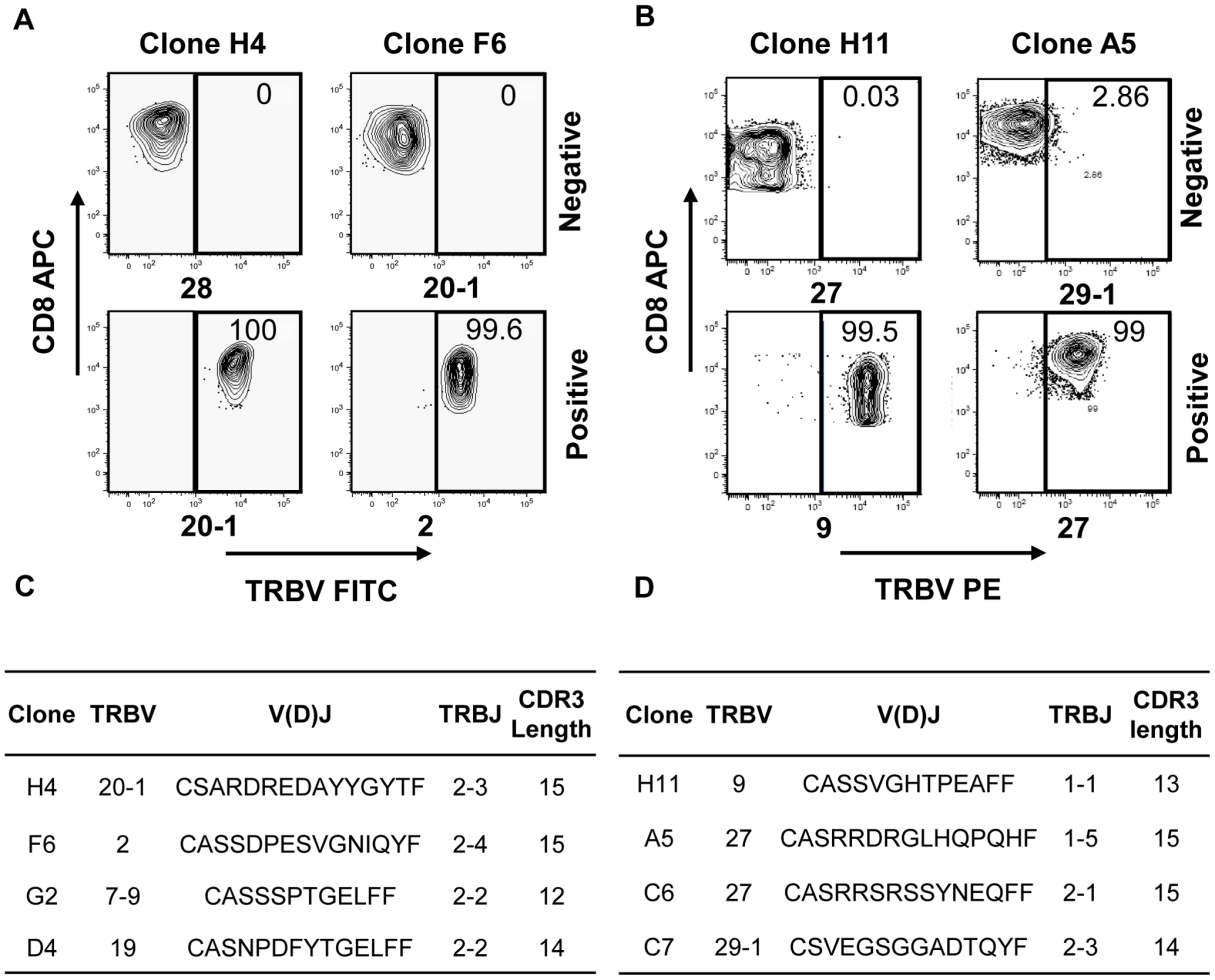
TRBV analysis of LCL- and tumour-reactive CD8+ T cell clones. CD8+ T cell clones specific for (A, C) LCL or (B, D) tumour antigens were assessed for clonality by flow cytometric and molecular analysis. (A, B) Clones were stained with a panel of fluorescently-labelled TRBV mAb, then analysed on a flow cytometer. Positive and negative TRBV staining is shown for representative T cell clones. (C, D) To confirm the flow cytometric findings and determine unidentified TRBV regions, RNA was extracted from T cell clones and TRBV regions reverse transcribed, amplified, then sequenced. The designations TRBV and TRBJ follow the TCR gene nomenclature specified by IMGT [18].

To confirm that T cell cultures generated by single-cell sorting were truly clonal, we undertook TRBV expression analysis of eleven clones from the LCL sort, using a panel of 23 mAb specific for different TRBV (representative clones shown in Fig. 6A). Six clones stained uniformly with a single mAb: TRBV20, TRBV22, and TRBV3 (2 clones) and TRBV2 (2 clones). These six clones were not stained by the other 22 TRBV mAb. The clones for which a TRBV was not identified (*i.e.* they were not stained by any of the 23 mAb) likely express TRBV that were not part of the mAb set. These data definitively support the clonality of these cultures, and show that feeder cells do not interfere with the analysis. Flow cytometric data was confirmed by sequencing the TRBV regions of these clones (Fig. 6C), and both forms of analysis were repeated on tumour antigen-specific T cell clones (Fig. 6B, D). All T cell clones stained positively with a maximum of one TRBV mAb and only one TRBV region was detected in T cell clone RNA, therefore we conclude that the generated T cells were clonal.

## Discussion

Here we describe a rapid and highly efficient method for cloning effector CD4+ and CD8+ T cells *ex vivo*, which can use a range of antigen sources as stimuli, and generates T cell clones with diverse specificities. The method enables the selection of T cell clones with known function *ex vivo*, without the need for prior, multiple rounds of *in vitro* stimulation and cell division. Subpopulations of T cells <0.1% above background (Fig. 1) were cloned and expanded to large numbers, and the capability of recording *ex vivo* phenotypic characteristics enables the linkage of these attributes with data subsequently generated *in vitro*.

Current cloning methods do not allow precise cloning efficiencies to be calculated, since limiting dilution analysis frequently underestimates the precursor frequency [22]. In the case of known epitopes, multimer technology does allow precursor frequencies to be estimated. Dunbar *et al*. sorted single HLA-A2-restricted, melanoma-epitope tetramer+ CD8+ T cells and cloned them at an average efficiency of 6.5% [9]. In contrast, the method described in this paper generates CD4+ and CD8+ clones without prior knowledge of epitope specificity or limitation of particular HLA types, and is thus suitable for goals such as antigen discovery and the analysis of broad responses to antigen. Since flow cytometric and sequence analysis of TRBV regions of the clones indicated that each clone had indeed originated from a single precursor, the method provides a direct estimate of cloning efficiency. Clonal expansion was successful for an average of 30% (CD4+) and 48% (CD8+) of effector T cells that were sorted from PBMC in response to autologous melanoma cells, LCL or a CMV protein. The proportion of clones that retained antigen-specific functionality varied widely among donors, and between CD4+ and CD8+ subsets. Although this could reflect the sorting and clonal expansion of IFN-γ false-positive (e.g., cross feeding) contaminants, the high functional capacity of CD8+ T cells cloned from melanoma patient D14 (despite a low precursor frequency) indicates that instability of functional characteristics likely contributes to this phenomenon. Importantly, the method described herein could be used to investigate this further. For example, index sorting data reveal that effector CD8+ T cells producing low amounts of INF-γ in response to CMV pp65 have a significantly lower cloning efficiency, conceivably due to a lessened fitness of T cells to replicate or to survive. It would be of interest to investigate whether this characteristic has clinical implications.

T cells are often cloned after cultures of PBMC are stimulated and allowed to expand *in vitro* [10], [23], [24]. This prevents the analysis of some precusor characteristics, based on cell surface marker or cytokine expression, due to the differentiation of cells within the *in vitro* cultures. The method described in this paper enables highly efficient selection of subpopulations of interest, by avoiding this extended *in vitro* culture step. Short-term stimulation with antigen limits the opportunity for T cell replication, and phenotypic and functional modulation, and also enables direct quantitation of the T cells of interest. Index sorting allows the “history” of each T cell clone to be examined retrospectively, allowing connections to be made between *ex vivo* and *in vitro* T cell characteristics. This becomes important when assessing parameters used to define T cell characteristics *in vivo* [25], [26], which may not be stable *in vitro*, e.g. CD27 and CD45RA.

By removing the need for extended stimulation with antigen and antigen-presenting cells, T cell clones can be generated rapidly. Clones are available for use within three weeks of sorting, and can be expanded to large numbers (10^7^–10^8^) after a further three weeks of culture. There is no need for re-cloning, which is often required following limiting dilution cloning. In addition, by avoiding the selective outgrowth of more rapidly dividing cells, direct cloning likely enhances the repertoire diversity of the resulting collection of clones.

The addition of exogenous IL-2 or a co-stimulatory antibody against CD28 did not noticeably improve the *ex vivo* response to melanoma cell stimulation in preliminary studies, but the proportion of T cells activated might, however, be enhanced through the use of these additional signals when using other antigen sources, or by using other growth factors e.g. GM-CSF [27], [28].

Although our cloning technique as described here is based on IFN-γ secretion, the method could be adapted to select T cells based on production of other cytokines for which secretion assays are available, such as TNF, IL-2 or IL-17, alone or in combination. We sorted directly from stained PBMC, but commercially available cytokine capture assays allow magnetic enrichment of extremely low precursor numbers, so the extension of our method to very rare subsets would be straight forward. Direct cloning might also be applied to other non-lethal methods of identifying antigen reactive T cells, such as membrane-bound TNF [29] and the expression of a wide variety of activation markers (reviewed in [30]). However, the reliance of direct cloning on effector function makes it unsuitable for expanding naïve precursors, which is achievable by limit dilution cloning of amplified T cells [31].

T cell clones generated using direct cloning have advantages for some specific applications that are of potential clinical importance. The method preferentially selects effector-memory T cells, with immediate functional ability, which presumably reflects the active, circulating anti-pathogen or anti-tumour population. Since the cloning efficiency is high, the application of clones to defining the antigenic repertoire or discovery of novel antigens [32] would enable establishment of an immunodominance hierarchy for identified epitopes. Furthermore, by sequencing T-cell receptors of clones recognising an epitope of interest, the level of diversity within the effector response can be determined.

In conclusion, the ex vivo effector cell cloning method described here provides a rapid, powerful and effective method of deriving antigen-specific T cells clones with traceable in vivo precursor function and phenotype, thus improving on functionality and applicability compared to traditional T cell cloning techniques.

## Supporting Information

Figure S1 **(A) Index sorting was used to assign** ***ex vivo*** **IFN-γ secretion levels (arbitrary fluorescence units), in response to CMV pp65, to individual CD8+ T cells seeded into wells, which were scored for subsequent growth into long-term clones (“Grew”** ***vs*** **. “Died”).** (B) Percentile plot showing cumulative percentage of cells that established clones (– –) or died (––) according to IFN-γ production. The distribution of IFN-γ production differed significantly between the two groups of cells (Kolmogorov-Smirnov test; P<0.0001).
(TIF)

Figure S2 **CD4+ clones derived from EBV stimulation were tested for proliferation in response to the autologous LCL by tritiated thymidine incorporation; the background proliferation (Nil stimulation) of a random selection of clones was also assessed.** Data indicate means of duplicate measurements in a single experiment. The geometric mean (––) and upper 99% confidence limit of the geometric mean (---) of proliferation of unstimulated clones are indicated.
(TIF)
