# Supplementary figures and images for: High Efficiency Ex Vivo Cloning of Antigen-Specific Human Effector T Cells
Source: PLoS One. 2014 Nov 4;9(11):e110741. doi: 10.1371/journal.pone.0110741 (PMC4219695; doi:10.1371/journal.pone.0110741)

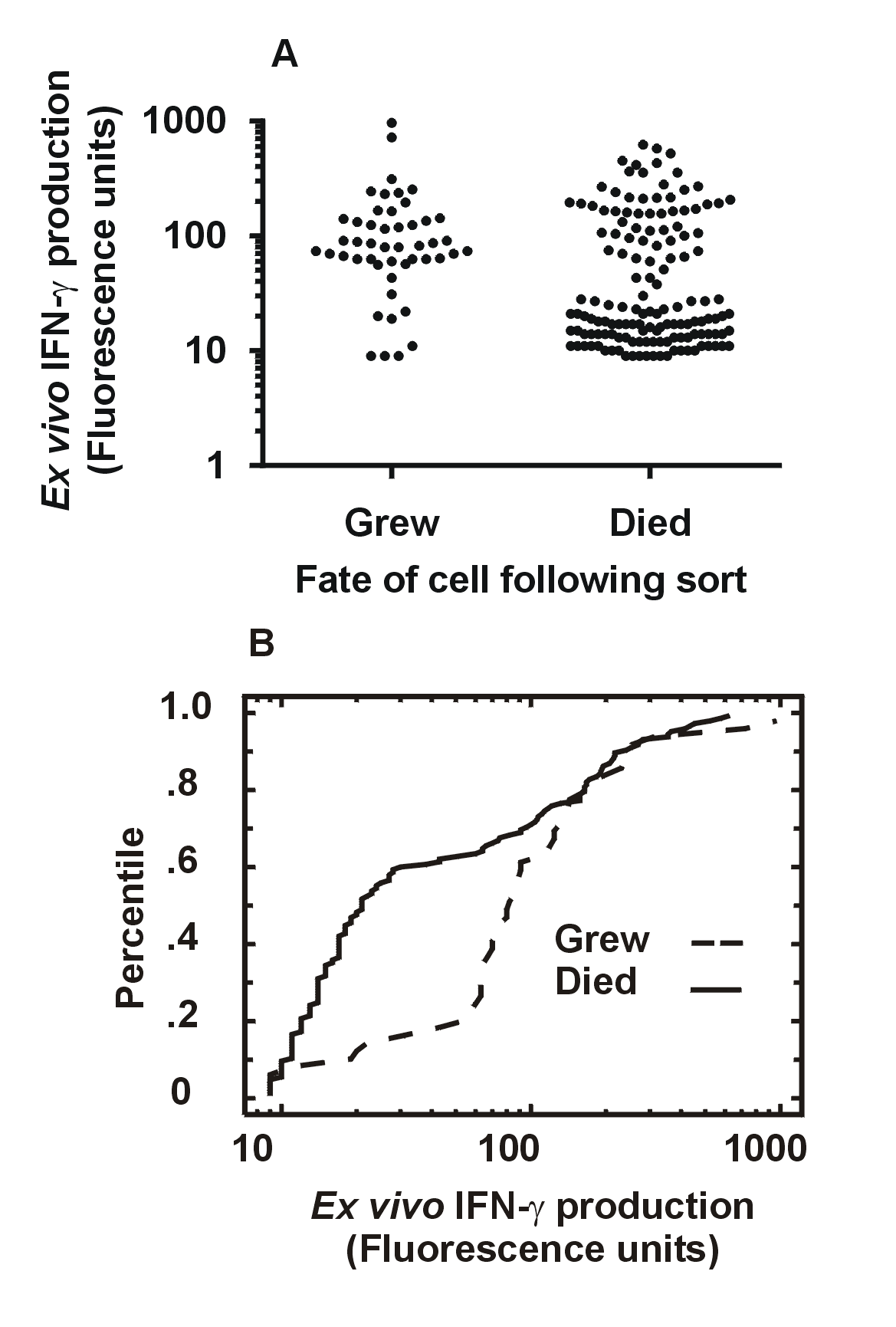

Supplement: Figure S1 — (A) Index sorting was used to assign ex vivo IFN-γ secretion levels (arbitrary fluorescence units), in response to CMV pp65, to individual CD8+ T cells seeded into wells, which were scored for subsequent growth into long-term clones (“Grew” vs . “Died”). (B) Percentile plot showing cumulative percentage of cells that established clones (– –) or died (––) according to IFN-γ production. The distribution of IFN-γ production differed significantly between the two groups of cells (Kolmogorov-Smirnov test; P<0.0001). (TIF) [file pone.0110741.s001.tif]

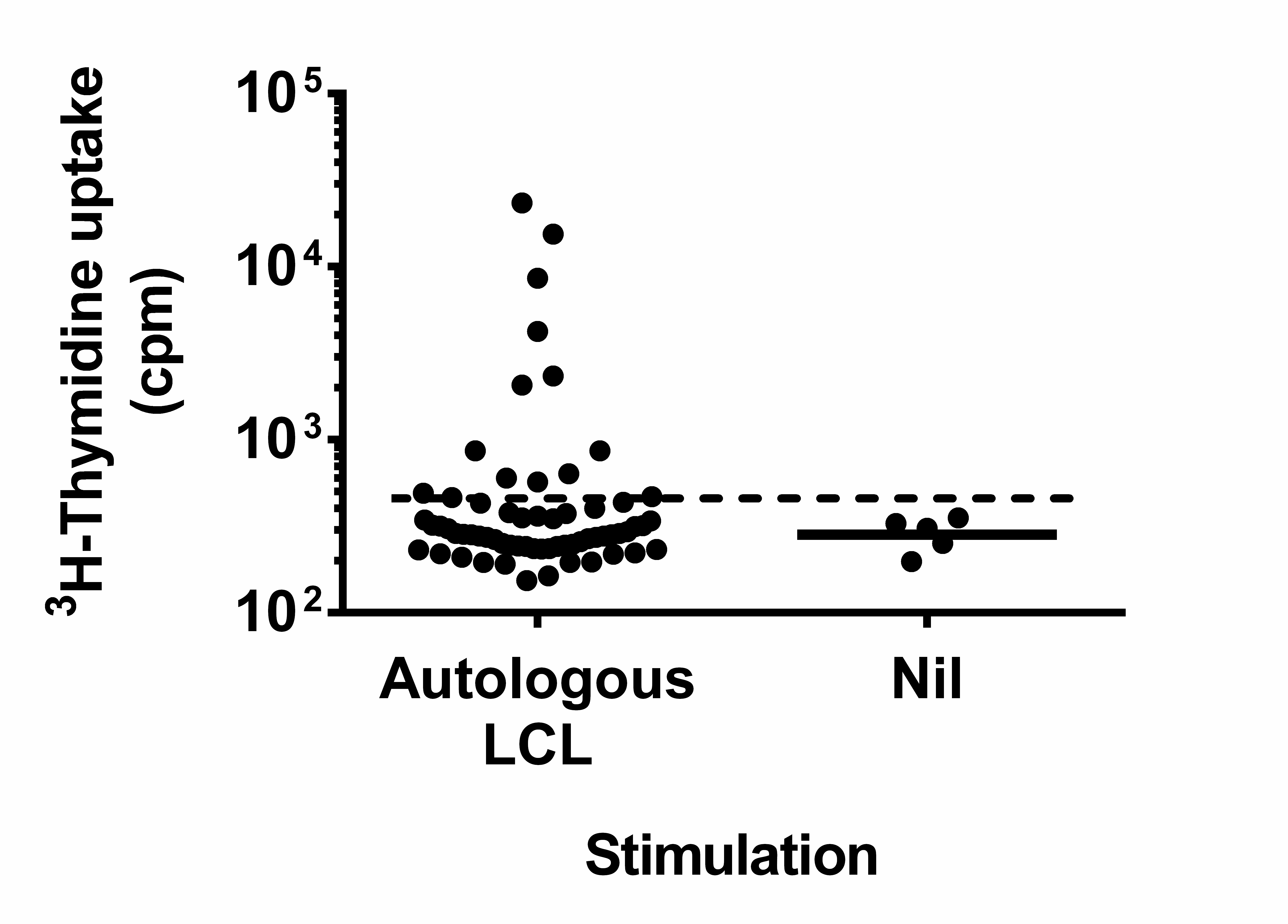

Supplement: Figure S2 — CD4+ clones derived from EBV stimulation were tested for proliferation in response to the autologous LCL by tritiated thymidine incorporation; the background proliferation (Nil stimulation) of a random selection of clones was also assessed. Data indicate means of duplicate measurements in a single experiment. The geometric mean (––) and upper 99% confidence limit of the geometric mean (---) of proliferation of unstimulated clones are indicated. (TIF) [file pone.0110741.s002.tif]
